# One-step synthesis of gold and silver non-spherical nanoparticles mediated by Eosin Methylene Blue agar

**DOI:** 10.1038/s41598-019-55744-0

**Published:** 2019-12-18

**Authors:** Diego Alberto Lomelí-Rosales, Adalberto Zamudio-Ojeda, Sara Angélica Cortes-Llamas, Gilberto Velázquez-Juárez

**Affiliations:** 1 Departamento de Química, Centro Universitario de Ciencias Exactas e Ingenierías, Universidad de Guadalajara. Departamento de Química. Blvd. Marcelino García Barragán #1421, C.P, 44430 Guadalajara, Jalisco México; 2 Departamento de Física, Centro Universitario de Ciencias Exactas e Ingenierías, Universidad de Guadalajara. Departamento de Química. Blvd. Marcelino García Barragán #1421, C.P, 44430 Guadalajara, Jalisco México

**Keywords:** Nanoparticles, Nanoparticles

## Abstract

Nowadays, there are several approaches reported to accomplish the green synthesis of metal nanoparticles by using bacterial and fungi supernatants or by-products generated by these microorganisms. Therefore, agars as solely reductive regents have started to be used in order to obtain metal nanoparticles. This paper shows the results of the synthesis of gold and silver nanoparticles with different morphology, mainly triangular and truncated triangular, using Eosin Methylene Blue (EMB) agar as reducing agent. To control the reaction process, the necessary activation energy for the reducer was provided by three different techniques: microwave radiation, using a domestic microwave oven, ultraviolet radiation, and heating on a conventional plate. The evolution of the reduction process and stability of the samples was performed by ultraviolet visible spectroscopy. Morphology was carefully analyzed using high-resolution transmission electron microscopy (HRTEM) and Transmission electron microscopy (TEM). A one step synthesis for gold and silver nanoparticles was optimized with an eco-friendly and economic process.

## Introduction

Metal nanoparticles (MNPs) are being extensively studied due to their unique properties and their wide range of applications. These properties depend on the chemical composition, size, dispersity, and controlled morphology and the medium where the nanoparticles are obtained^1^. In addition to these requirements, a great interest has emerged on the synthesis of NPs using the principles of green chemistry^2^. One of the biggest challenges within nanoscience is the control of the fore mentioned characteristics during the synthesis of NPs while an environmentally friendly process is performed^3^. Development of such strategies have the potential to eliminate the use of common organic solvents, aggressive reducing agents (such boron hydrides) or the addition of polymeric stabilizers; improving the biological compatibility of NPs and their application in biology, medicine and green catalysis^4–6^. Nowadays, there are several approaches reported to accomplish the green synthesis of NPs by using plant extracts^7,8^, different sources of microbial biomass^9^, formulations based on honey^10^ among others^3,11^. Besides the reduced impact to the environment, green synthesis of nanoparticles it is usually simple, less expensive and the size and optical properties comparable to those obtained by conventional methods^12,13^.

In addition to the green processes that are booming, it is also necessary to find new ways to control the morphology of MNPs. So far, there are some reports where agents like long chain quaternary ammonium salts, or surfactants are used in order to modify spherical MNPs morphologies to nanowires, nanostars and even nanoprisms. By the other hand, seeding processes has been used to improve non spherical morphologies in MNPs^14^. Even though both strategies mentioned above involve longer and more complicated synthesis and the yield is too low^15–17^.

Nowadays, there are several approaches reported to accomplish the green synthesis of NPs by using: extracellular or intracellular extracts of thermophilic filamentous fungi^18^, *Trichoderma viride*^19^ filtrate, glycolipids from *Lactobacillus casei*^20^ and other bacterial culture media supernatants^21^. Although experimentation with agars in order to obtain metal nanostructures had begun to be implemented^22^, there is still several methodological strategies unexplored using agars as solely reducing and stabilizing agent. Agars are mixtures of compounds that are typically used for microorganism’s growth. Some of the agar components are susceptible to oxidation, which allows them to be used as reducing agents, these characteristics opens an interesting field of study to assess the capacity of agars to reduce metals and therefore generate nanoparticles. At the same time, agars are fully biodegradable and environmentally friendly. In order to contribute to new methodologies for synthesis of nanostructures using the principles of green chemistry and the generation of practical approaches to control MNPs morphologies.

This work explored the potential of Eosin Methylene Blue (EMB) agar as an exclusive reducing agent for synthesis of MNPs. Synthesis of gold (AuNPs) and silver (AgNPs) nanoparticles through the using of EMB agar was achieved. The activation energy for the control of the reaction process, was provided by three different techniques: microwave radiation, ultraviolet radiation, and heating on a conventional plate with stirring. Evolution of the reduction process and stability of the samples was performed using ultraviolet visible spectroscopy. Morphology was carefully analyzed using high-resolution transmission electron microscopy (HRTEM) and Transmission Electron Microscopy (TEM). A one step synthesis approach was developed for the production of MNPs with predominantly non-spherical morphologies such triangular and truncated triangular shapes.

## Experimental Section

### Materials

All chemicals were used without further purification. Silver nitrate (AgNO_3_) and chloroauric acid trihydrate (HAuCl_4_·3H_2_O) were obtained from Sigma Aldrich. Eosin methylene blue (EMB) agar, was provided by MCD-LAB Mexico.

### Characterization

Morphology and size of the MNPs were characterized using a Jeol JEM 2100 instrument, operated at 200 kV for HRTEM/EDS and 90 kV for TEM. UV-vis absorption spectra were recorded at room temperature using a Thermo Scientific Genesys 10 S spectrometer, the UV-vis spectra were recorded over the range of 200–1000 nm by a UV- VIS.

### General synthesis of gold and silver nanoparticles

One gram of EMD agar was dissolved in 100 mL of hot water (∼90 °C) distilled water and filtered by Whatman general purpose paper. Then, five milliliters of filtered solution were mixed with 1.0 mL of 8.0 mM of AgNO_3_ or HAuCl_4_. In order to promote metal reduction, solutions of agar and metal ions were subjected to three different treatments: (a) heating between 40–90 °C, (b) radiation with UV light during 15 to 60 minutes and (c) radiation by microwave during 5–15 seconds at 500 Watts.

EMB broth was prepared using all the components at the same concentration it would have if one gram of EMD agar were dissolved in 100 mL but without the agar component (lactose and sucrose: 0.14 g, peptone: 0.28 g/, dipotassium phosphate: 0.06 g methylene blue 0.002 g and eosin 0.011 g, all components dissolved in 100 mL of water). Eosin and Methylene blue solutions were prepared at 0.011 g/100 mL and 0.002 g/100 mL respectively. Experiments were performed as describe above, the only difference was the use of EMB broth, MB solution or eosin solution instead of EMB agar.

## Results and Discussion

### Synthesis of MNPs by Eosin Methylene Blue medium (UV-EMB method)

**Figure 1:**
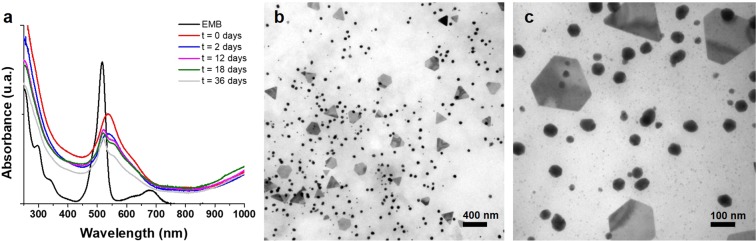
UV-VIS absorption spectra for AuNPs. Spectra was measured at different times after being synthesized, demonstrating the stability of the nanostructures in solution. (**b**) TEM image of a gold nanoparticles sample, where it is possible to observe that there is a homogeneous distribution of triangular nanoparticles. (**c**) A zoom of the figure b, where it is possible to observe a third kind of particles with diameters smaller than 10 nm.

In the first series of experiments, synthesis of nanoparticles was conducted by continuous UV light radiation in EMB solutions containing the corresponding metal salts (AgNO_3_ or HAuCl_4_). To promote the reduction of metal ion and oxidation EMB solution, a UV light lamp was used (20 minutes of exposition was enough to produce AuNPs at 30 °C). Figure 1a, shows the results of AuNPs obtained at 20 minutes of UV exposure (day 0) compared with the EMB solution media, it can be observed that the EMB solution presents two bands, in the ranges from 450 to 550 nm and from 600 to 750 nm; the first one belongs to a narrow and intense band while the second is broader and less intense. Contrastingly, day-0 AuNPs, presents a 25 nm shift in the first band which is less intense and broader in the range of 475 nm to 700 nm; while the second band shows a bigger shift that is inferred by the rising of the baseline starting at 800 nm, suggesting that the maximum band absorption is placed at the near IR spectra, several authors^23–25^ have reported that this rising is an indicative of a non-spherical morphology when presented in AuNPs spectra. Stability of nanoparticle solution was monitored up to 36 days by UV-VIS scanning, spectral curves of aged nanoparticles (2, 12, 18 and 36 days) did not show any perceptible changes in shape or intensity. TEM images confirmed the non-spherical shape of nanoparticles obtained by the UV-EMB method. The morphology of nanoparticles is presented in Fig. 1b,c. On the basis of the TEM images, faceted morphologies were observed for nanoparticles, three different group of sizes can be differentiated, the largest are basically formed by triangular and triangular truncated shapes. The medium size with a faceted non regular form, and the smallest that apparently showed semispherical shapes (Fig. 1b,c).

**Figure 2:**
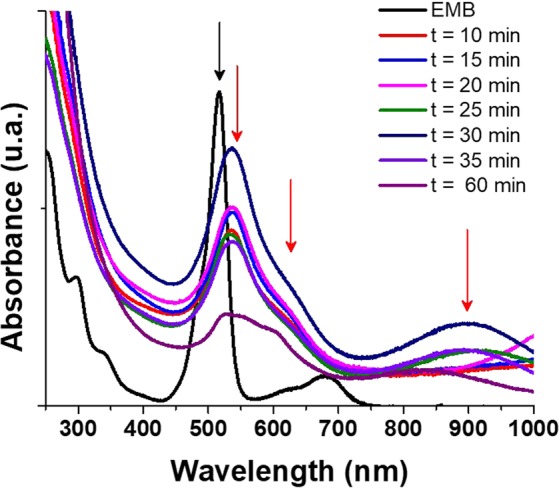
UV-vis spectra for AuNPs at different exposure times to UV radiation. The red arrows remark the principal absorption curves for the obtained AuNPs, which allows to observe with greater clarity the differences in the second absorption curve with better detail around 900 nm approximately.

By other hand, an experiment was performed to determine the effect of UV exposure time during the synthesis of AuNPs. It seems that UV incidence time is an important parameter when synthesis of AuNPs is performed by UV-EMP method. Figure 2 (magenta line) shows that at the exposure time of 60 min the UV spectra of NPs change dramatically and in experimental conditions a deposition of macroscopic Au^0^ was observed (evidenced by the change in the color of colloidal solution form purple to black and a golden mirror observed on the walls of the vial). Not only the stability of AuNPs is affected by the radiation time but the size of nanostructure obtained. Figure 2 shows UV-vis absorption spectra for AuNPs with UV exposure times from 10 to 60 minutes. It can be observed that with 10 minutes of exposure, there is already nanoparticles presence (evidenced by shifting in the absorption bands similar to those described in Fig. 1a). Through this analysis it was found that after 25 minutes of radiation, the size of the AuNPs diminished considerably; this was inferred by the hypochromic shift presented in the region around 925 nm (when compared with Fig. 1a red line). The same effect was observed for the samples irradiated at 30 and 35 minutes (presenting a very defined band around 900 nm). The optimal time of UV exposition was found to be 30 minutes for the AuNPs synthesis; with maximum hyperchromic effect (equivalent to maximum concentration of AuNPs in solution). This AuNPs are stable up to one month without any significant changes in morphology or sizes (UV spectra are presented in supplementary material).

**Figure 3:**
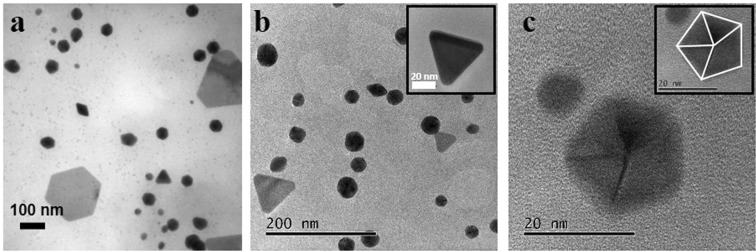
TEM images for AuNPs at different exposure times to UV radiation: (**a**) 20 minutes, (**b**) 30 minutes and (**c**) 60 minutes.

Figure 3 presents the TEM images obtained for AuNPs samples obtained at 20 (a), 30 (b) and 60 (c) minutes of UV radiation time, it is possible to corroborate that the size of AuNPs is related to the time of exposition. The AuNPs size is decreased as radiation time is increased.

AgNPs were synthesized in a very similar way to AuNPs synthesis mentioned above, although a time of 10 minutes of UV light exposition was enough to reduce silver to AgNPs, there was no change in morphology or precipitation within the 60 minutes of exposition for the synthesis according to observed curves in the UV-vis spectra (see supplementary information Fig. 1).

**Figure 4:**
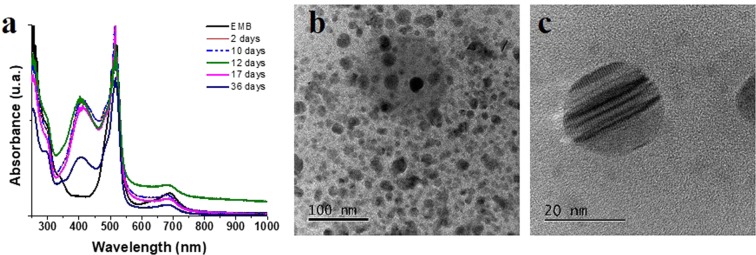
UV-vis absorption spectrum of the AgNPs, where an intense band is observed at 400 nm corresponding to its plasmon (**a**) and its corresponding TEM image where the formation of hemispheres was observed (**b**,**c**).

**Figure 5:**
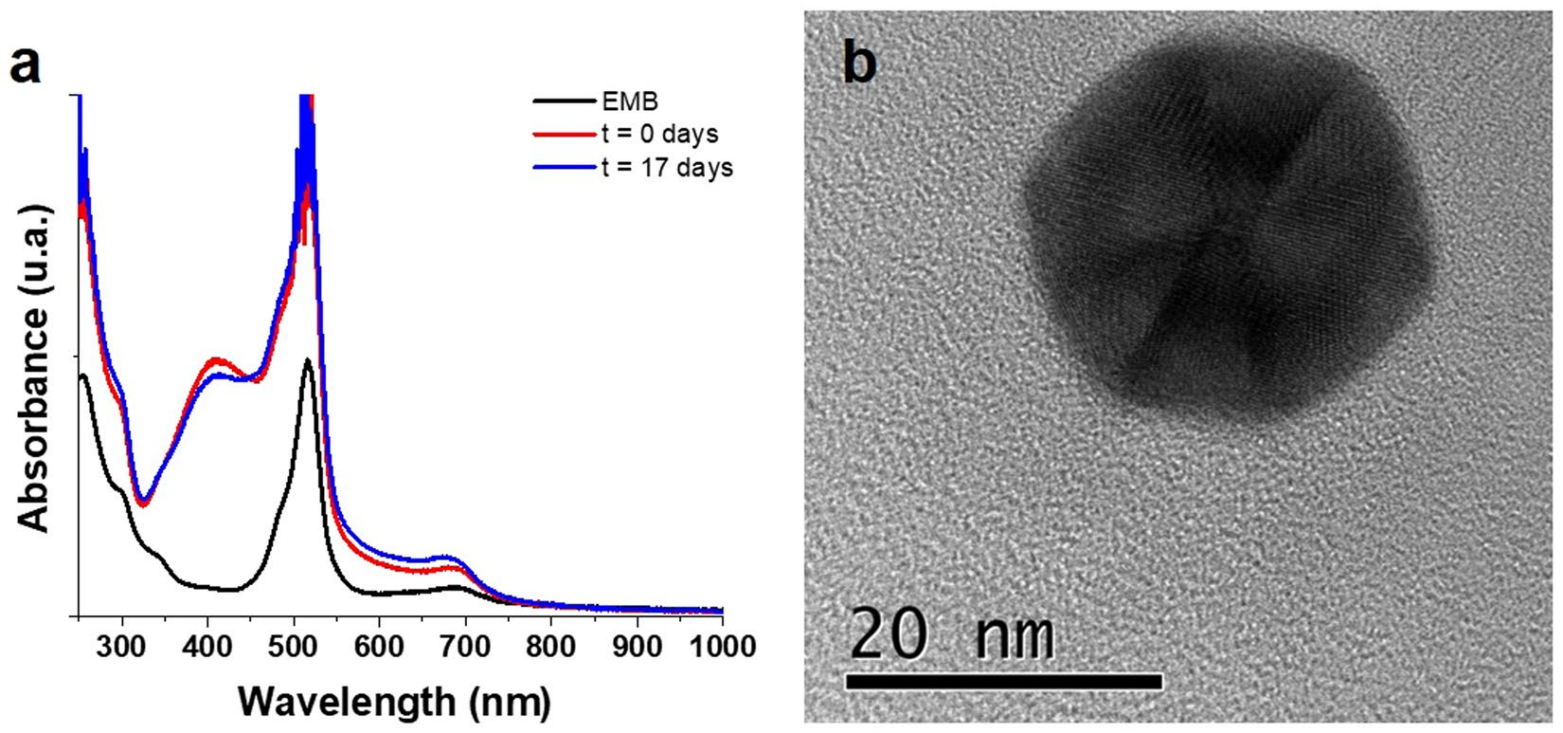
UV-vis absorption spectrum of the AgNPs at 3 hours of radiation, where a band is observed at 400 nm, less intense than for those at times lower than 40 minutes of exposure (**a**). TEM image where the formation of faceted icosahedra was observed (**b**).

Figure 4a shows the UV-Vis absorption spectrum of the AgNPs obtained, where an intense curve is observed at 400 nm, which is an indicative of AgNPs formation. Unlike the AuNPs, the absorption bands observed for EMB solution are preserved during the process, and there was no presence of 900 nm band indicating a non-spherical morphology. Nevertheless, hemispherical morphologies were observed through TEM (Fig. 4b,c). Similar to AuNPs, a stability study of the AgNPs in solution was carried out, finding great stability on the part of these nanostructures (up to one month after being synthesized NPs still remain in solution). A determination of the optimal exposition time was evaluated (supplementary information), and a special time of overexposure (3 hours) to UV radiation was performed. Interestingly, a prolonged exposure of the UV radiation to the AgNPs did not result in the precipitation of these or the reaction medium, although a variation in morphology was observed. With the overexposed UV treatment, it was feasible to obtain nanostructures of icosahedrons of approximately 15 nm of edge (Fig. 5b,c). In addition, it was possible to observe the phases in the nanostructure (Fig. 5c), indicating that it was not the result of a controlled nucleation, but rather, of a nucleation in parts (Fig. 5). These nanostructures were stable up to 17 days.

For a better understanding of the reducing power of this medium, MNPs were synthesized by thermal treatment. Solutions of agar and metal ions were heated at 40, 60 and 90 °C, to evaluate the influence of temperature during the synthesis of nanoparticles.

**Figure 6:**
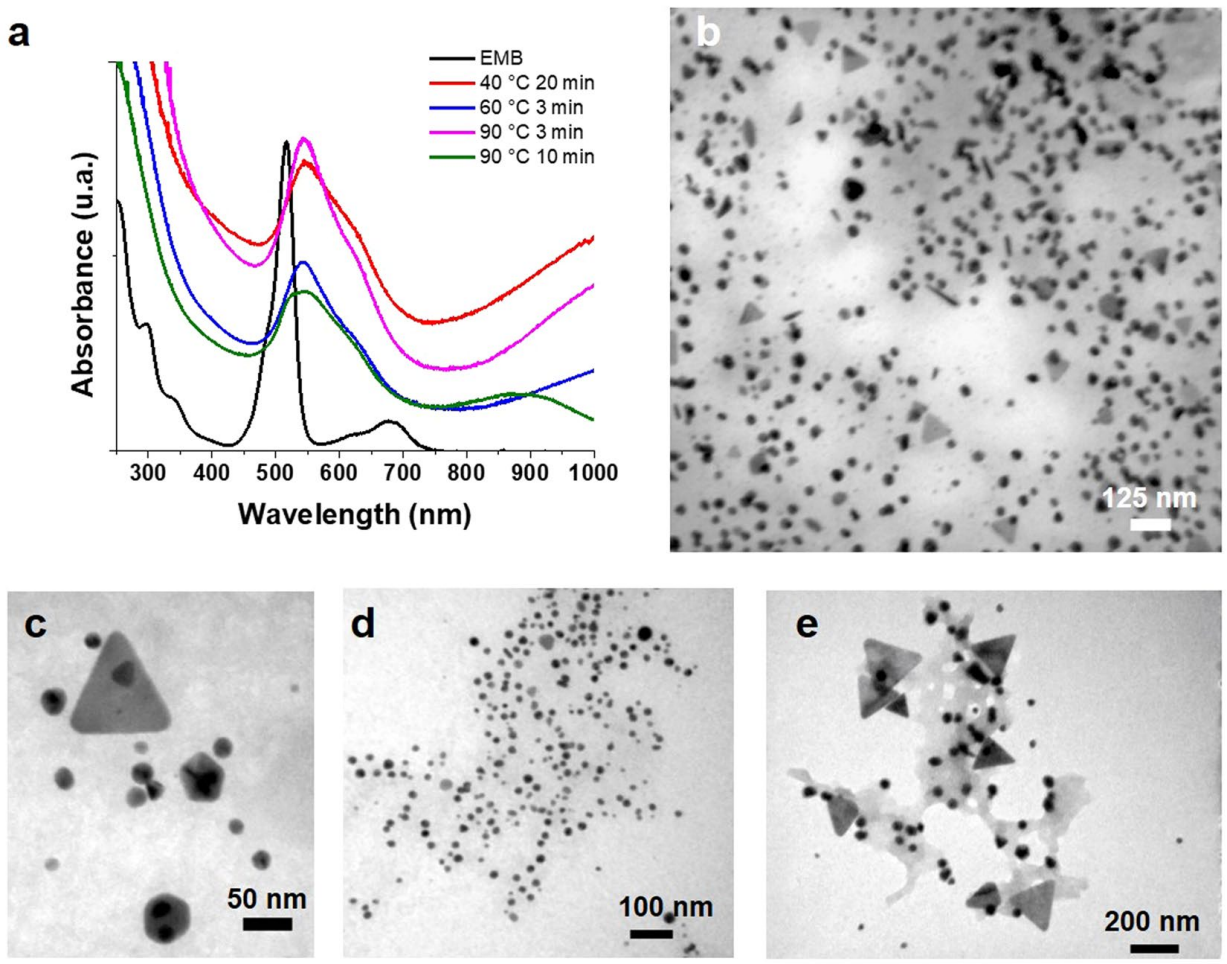
UV-vis absorption spectrum of the AuNPs at different temperatures and reaction times (**a**), TEM images for different times and reaction temperatures: 90 °C and 3 min (**b**); 90 °C and 10 min (**c**); 60 °C and 3 min (**d**); 40 °C and 20 min (**e**).

UV-Vis spectra for the AuNPs synthesized at 90 °C are shown in Fig. 6a, a similar behavior in the spectral curves obtained with UV radiation is clearly observed. At 90 °C of temperature, formation of nanoparticles was very fast, fundamentally when the vial was immersed in the heated oil bath, the solution started to color immediately, and the reaction was stopped (by cooling at RT) after 3 and 10 minutes for further analysis. At 60 °C, the reaction occurred as fast as 90 °C while, at 40 °C the reaction proceeded more slowly, (taking up to 20 minutes to complete the reduction). These results indicate that this system is very sensitive to temperature increments. The resulting predominant morphologies of AuNPs were among the spherical small sizes morphologies to equilateral triangles of about 50 nm of edge (Fig. 6b–e). As previously observed with UV radiation, the process, at longer times of synthesis, smaller sizes were obtained.

**Figure 7:**
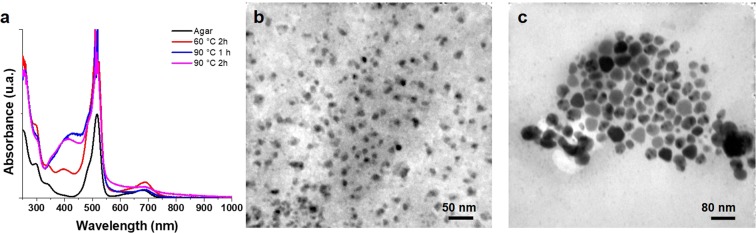
UV-vis absorption spectrum of the AgNPs at different temperatures and reaction times (**a**), TEM images for different times and reaction temperatures: 90 °C and 1 h (**b**); 60 °C and 2 h (**c**).

When reproducing the thermal experiments to obtain AgNPs, the results were completely unexpected, because the reaction times were longer than previously thought. While for AuNPs synthesis, the incubation time was between 3 to 20 minutes, for AgNPs synthesis it turned out to need times between 1 to 2 hours at 90 °C (for which a reflux system was placed to avoid water loss due to evaporation). Spectral UV-VIS curves for the synthesis of AgNPs shows that 2 hours and 90 °C are the best conditions to perform nanoparticle synthesis with silver in thermal process (Fig. 7a). The obtained morphologies were spheres with sizes between 9 to 10 nm approximately. For the synthesis at 60 °C and 2 hours of reaction, AgNPs were also observed, however, these solutions were diluted, as can be seen in the absorbance of its curve at 400 nm and which was subsequently corroborated by TEM (Fig. 7c).

**Figure 8:**
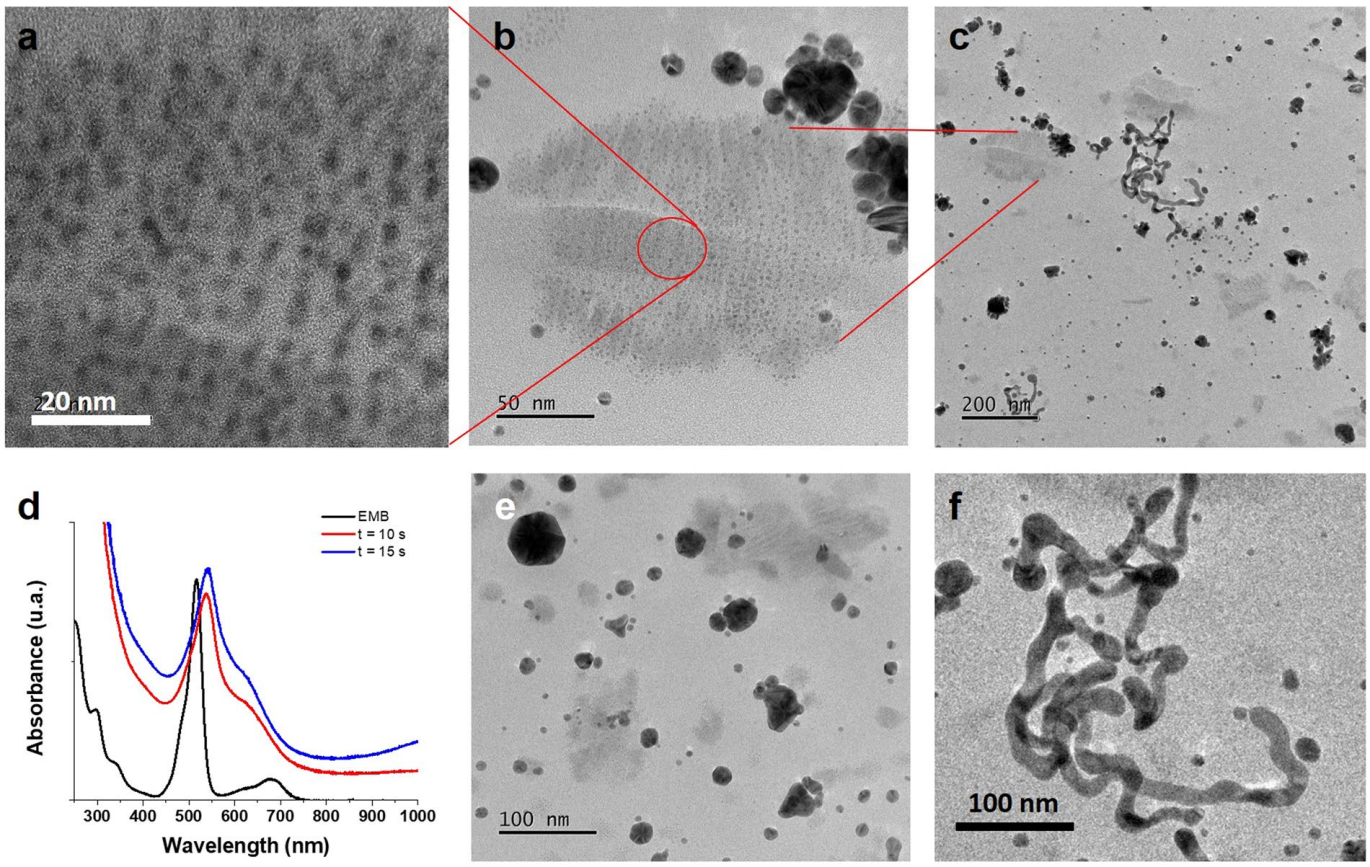
TEM images for AuNPs with 15 seconds of microwave synthesis. (**a**) Represents an enlargement of b, where a kind of association between the smaller AuNPs were observed, while (**b**) represents the increase of the image (**c**) to observe the actual size of said AuNPs of approximately 4 nm in diameter.

Therefore, we proposed to synthesize MNPs through the use of EMB solution and a microwave oven, using a domestic microwave oven, where at shorter reaction times, than those found for the methodologies previously described, were expected. For AuNPs formation it was established that the adequate reaction time was no greater than 15 seconds in a microwave at 500 watts (time and power were determined in order to avoid loss of solvent), due to longer times promoted the precipitation of the AuNPs from the reaction medium (a purple precipitate and a slightly colored solution was observed). The UV-Vis spectra of AuNPs showed one absorption band in the range of 500–700 nm and an inferred band in the near IR (Fig. 8d).

The images obtained by TEM for AuNPs synthesized by the microwave method showed 3 different morphologies: one of them being hemispheres around 40 nm (Fig. 8b,e), other being a very peculiar morphology spheres of less than 5 nm with tendency to associate (since these were aggregated among themselves, but isolated from the rest of the larger morphology spheres present) (Fig. 8a–c) and the third morphology that also called attention were a species of “twisted” nanowires or in “s” form, which may be responsible for the absorption curve in the near IR observed in the UV-vis spectrum (Fig. 8c,f).

However, implementing this same procedure to obtain AgNPs resulted in minimal formation, even when a power of a 100% was used with the microwave oven. The UV-vis absorption spectrum showed a slight absorbance curve at 400 nm, nevertheless this was with a highly concentrated sample, where the contribution of that curve is minimal, compared with the curves that have been obtained up to this point by the other methodologies, so for this case, this process was considered inefficient for AgNPs synthesis (Fig. 2 SI).

With the presented results throughout this work, it has been showed that EMB agar is a suitable reducing agent for the generation of MNPs. It is important to mention, that EMB agar contains high concentrations of lactose (wt% 27 approximately), which is considered as a reducing sugar; this means that an hemiacetalic hydroxyl group (–OH) (capable of been oxidized) is present. The abundance of this carbohydrate along with the adequate activation energy could be performing the metal reduction in solution, while some of the other components in the agar media (such phosphates and eosin) could be stabilizing the MNPs. It is important to mention that the components of the EMB agar not only allowed an excellent stabilization of the MNPs (demonstrated by the lacking of decomposition when obtained in optimal conditions) but also led to the obtention of various nanostructures which can be favored in size and shape by modification of reaction times or activation energy used.

**Figure 9:**
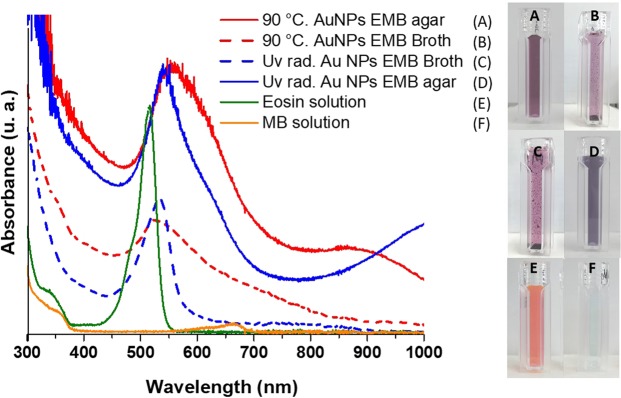
UV-VIS absorption spectra of the AuNPs produced by heat and UV radiation. Solid red line, 90 °C heat treatment with EMB agar, dashed red line, 90 °C heat treatment with EMB Broth. Solid blue line, UV radiation treatment with EMB agar, dashed blue line, UV radiation treatment with EMB Broth. Green line Eosin solution spectra and orange line methylene blue (MB) solution spectra. Images on the right side, show the color of the AuNPs obtained; AuNPs produced with EMB agar (**A**; heat treatment and **D**; UV radiation treatment). AuNPs produced with EMB Broth (**B**,**C**) where instability evidenced by precipitation is observed.

One of the original purposes of using EMB agar as a reducing agent was to take advantage of the fluorescence of the eosin component, we expected that this agent would be able to coordinate to the metal surface of NPs, providing fluorescence to it. However, once the MNPs were synthesized and isolated by centrifugation, the solid obtained did not show any fluorescence (data not shown). Therefore, we decided to study the role of some components of the EMB agar during the formation of AuNPs. A series of experiments were conducted using a solution with the components of EMB media without agar (solution that we referred as EMB Broth). Figure 9 shows the results of treatment by UV radiation and heat at 90 °C to the HAuCl_4_ solution with EMB broth and EMB agar. Eosin solution presented a unique and intense band with a maximum absorption at 516 nm while methylene blue (MB) solution presented a slight contribution at 663 nm. When EMB Broth and EMB agar experiments are compared a decrease in the intensity of the 530 nm band is observed with EMB Broth. Moreover, the absence of agar (agarose polysaccharide) caused AuNPs precipitation in both treatments: UV (Fig. 9C) and heat treatment (Fig. 9B). These experiments assess, that components of Broth are enough to cause reduction of the MNPs but the presence of agar in our experiments demonstrated not only the reducing power as previously reported^22^ but an important role for colloidal stability of MNPs. It is important to mention that when controls with MB solution + HAuCl_4_ and Eosin + HAuCl_4_ were subjected to UV-VIS scanning, AuNPs were not obtained (see supplementary information, Figs. 4 and 5).

## Conclusions

Silver and gold nanostructures with non-spherical morphologies were synthesized in a one-step methodology by using EBM agar components. The advantage of synthesis with EMB agar (as reducing and stabilization agent) is in the substitution of the conventional compounds that in most cases can turn out to be environmentally hazardous. The monitoring of samples by UV-VIS spectroscopy allowed us to confirm the stability of the nanostructures in solution; being especially notable that in some cases there were no changes in sizes and morphologies for at least one month.

When a comparison among synthesis exposed in this work was performed, it was clear that morphologies can be controlled by making variations on UV exposure times, as well as in sizes of the nanostructures in general. Nevertheless, if the energy provided for the reaction was by heating or microwave radiation; the nanostructures presented drastic changes; providing valuable information about the synthesis path, and the way energy used is directly related with the morphology and facets that nanostructures are formed.

The use of microwave is perhaps, in the case of AuNPs, the most interesting synthesis method, because when 10 seconds of exposure are reached, several peculiar nanostructures were obtained. Although when the exposure time is larger than 15 seconds it led to precipitation. In contrast, for the obtention of AgNPs, the microwave technique is not the most appropriate.

In summary, the optimal technique for the obtention of AgNPs using EMB agar is UV radiation and heating, while for AuNPs any of the three techniques favored the formation of the nanostructures. For all of the above mentioned, this work opens the possibility of studying this kind of systems for the generation of metallic nanostructures in solutions, in a one-step economical synthesis and with the possibility of getting different non spherical morphologies at the same time.

## Supplementary information

Supplementary information.
