# Supplementary material for: One-step synthesis of gold and silver non-spherical nanoparticles mediated by Eosin Methylene Blue agar
Source: Sci Rep. 2019 Dec 18;9:19327. doi: 10.1038/s41598-019-55744-0 (PMC6920435; doi:10.1038/s41598-019-55744-0)
Supplement: Supplementary file 1 — Supplementary information. [file 41598_2019_55744_MOESM1_ESM.docx]

**One-step synthesis of gold and silver non-spherical nanoparticles mediated by Eosin Methylene Blue agar.**

## 1 Diego Alberto Lomelí-Rosales (Departamento de Química, ORC ID - 0000-0002-9949-5829)

## 2 Adalberto Zamudio-Ojeda (Departamento de Física, ORC ID – 0000-0002-8684-193X)

## 3 Sara Angélica Cortes-Llamas (Departamento de Química)

## 4 Gilberto Velázquez-Juárez* (Departamento de Química, ORC ID - 0000-0002-1130-4021,

## *corresponding autor: [gilberto.velazquez@academicos.udg.mx](mailto:gilberto.velazquez@academicos.udg.mx), Phone: +52 (33) 1378 5900 ext: 27682

Centro Universitario de Ciencias Exactas e Ingenierías, Universidad de Guadalajara. Blvd. Marcelino García Barragán #1421, C.P. 44430, Guadalajara, Jalisco, México.


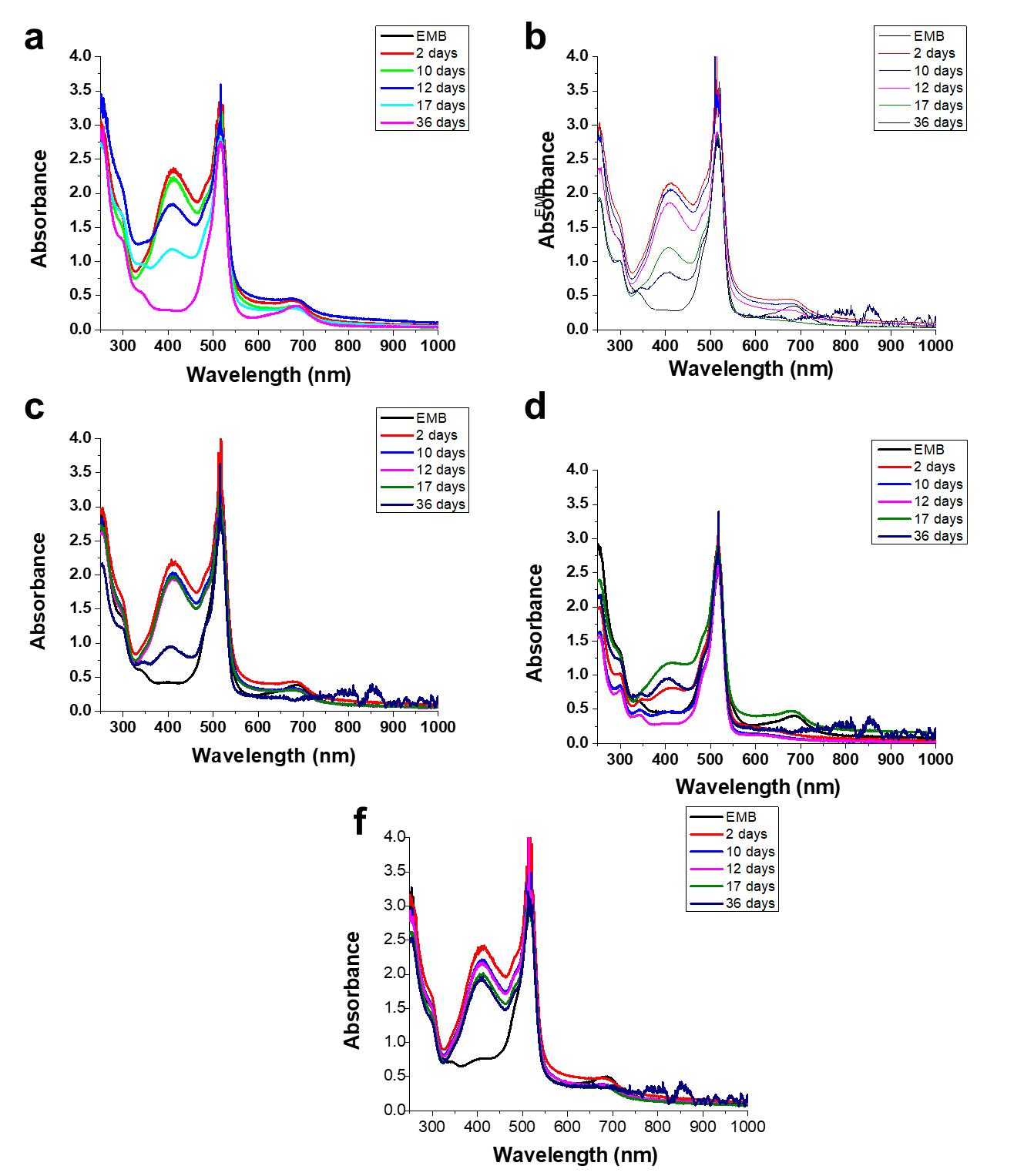


**Figure 1:** Spectrum of UV-vis absorption of the AgNPs at: a) 20 min, b) 30 min, c) 40 min, d) 50 and e) 60 min of radiation, where a band is observed at 400 nm.

**Figure 2:** UV-vis absorption spectrum for 1-NPsAg-M at different radiation microwave synthesis times.

**Figure 3:** UV-VIS spectra comparison of AuNPs with an unfiltered and filtered solution of EMB agar. In order to test that the filtration treatment described in the methodology, did not affect the production of MNPs a UV-VIS spectra was done demonstrating that there was no effect due to the filtration in the bands produce by the MNPs.


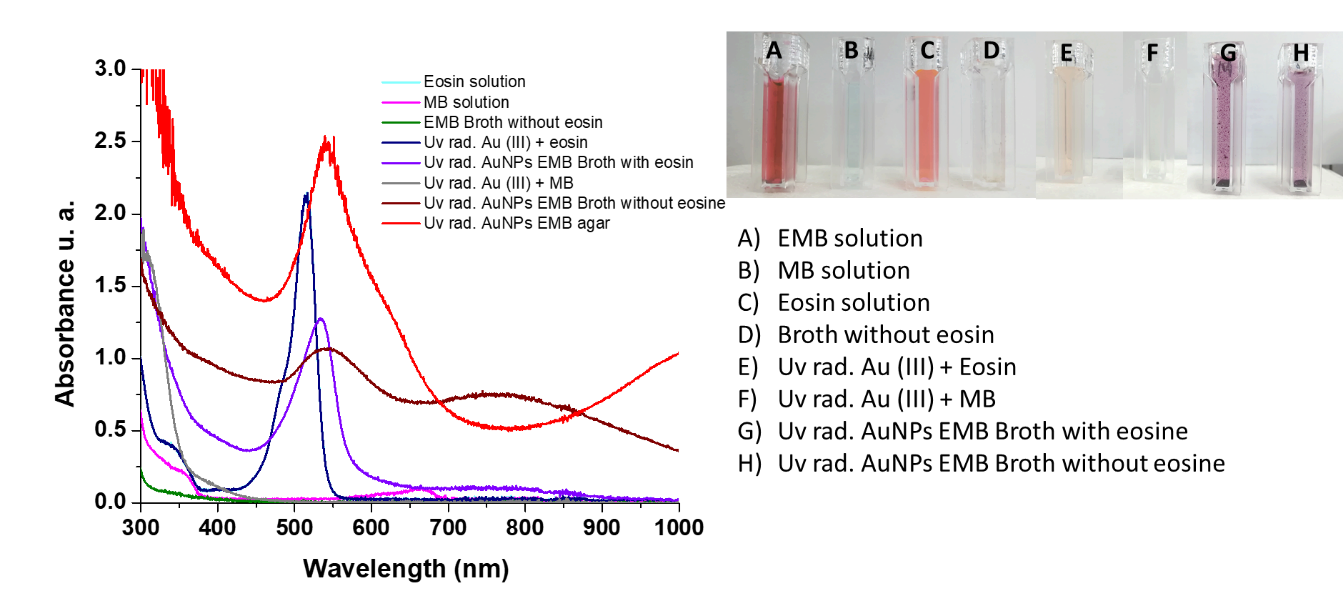


**Figure 4:** UV-Vis spectra of AuNPs obtained by UV radiation and Controls. To analyze the influence of each of the EMB agar component on the formation of the gold nanoparticles, the synthesis process was carried out using ultraviolet light, as a promoter of the reduction process. It is possible to observed the reduction of the ions was not carried out in eosin or in methylene blue. However, when the same process was carried out with EMB broth, a peak of maximum absorbance around 524 nm it was observed in the UV-vis spectrum, related to the formation of gold nanoparticles. The reduction effect could also be observed in the color change, figures G and and H, evidenced by purple color change.


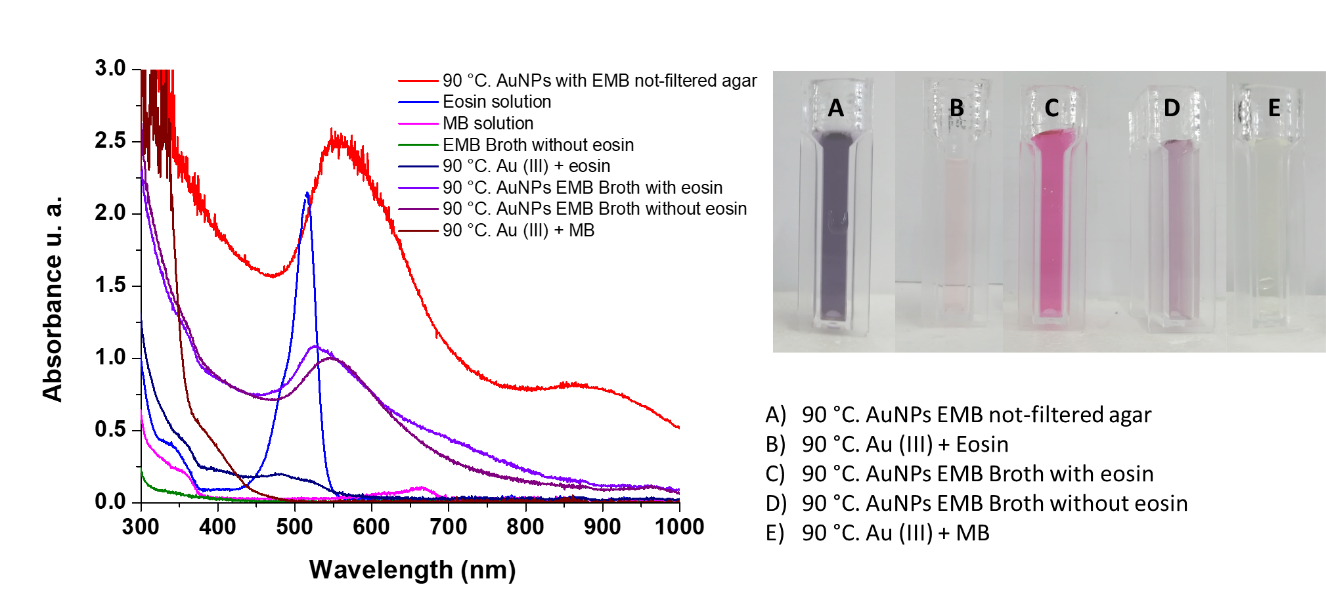


**Figure 5:** UV-Vis spectra of the AuNPs obtained by heat at 90º C and Controls. To analyze the influence of each EMB agar component on the formation of the gold nanoparticles, the synthesis process was carried out using heating in a hot plate at 90 ^ο^C, as a promoter for the reduction process. It is possible to observed the reduction of the ions was not carried out in eosin or in methylene blue. However, when the same process was carried out with EMB broth, a peak of maximum absorbance around 524 nm it was observed in the UV-vis spectrum, related to the formation of gold nanoparticles. Besides the right side images show the reduction evidenced by purple color change (A, C and D).

**Figure 6:** FT-IR- ATR of medium and the nanoparticles samples obtained by different treatment for synthesis. The spectrum shows that agar does not have any modification when the reduction of the ions is motivated by thermal treatments. However, when the reduction was promoted by a source of UV light the medium suffered a dramatically change evidence by the disappearance of the 3250 cm^-1^ band.
